# Stress-Related LncRNAs and Their Roles in Diabetes and Diabetic Complications

**DOI:** 10.3390/ijms26052194

**Published:** 2025-02-28

**Authors:** Lian Li, Yu-Qi Wu, Jin-E Yang

**Affiliations:** MOE Key Laboratory of Gene Function and Regulation, School of Life Sciences, Sun Yat-sen University, Xin Gang Xi Road 135#, Guangzhou 510275, China; lilian2@mail.sysu.edu.cn (L.L.); wuyq263@mail2.sysu.edu.cn (Y.-Q.W.)

**Keywords:** lncRNA, ER stress, oxidative stress, diabetes, diabetic complications

## Abstract

Diabetes mellitus (DM) is a chronic metabolic disorder and one of the most significant global health burdens worldwide. Key pathophysiological mechanisms underlying its onset and associated complications include hyperglycemia-related stresses, such as oxidative stress and endoplasmic reticulum stress (ER stress). Long non-coding RNAs (lncRNAs), defined as RNA transcripts longer than 200 nucleotides and lacking protein-coding capacity, play crucial roles in various biological processes and have emerged as crucial regulators in the pathogenesis of diabetes. This review provides a comprehensive overview of lncRNA biogenesis and its functional roles, emphasizing recent findings that link stress-related lncRNAs to diabetic pathology and complications. Also, we discuss how lncRNAs influence diabetes and its complications by modulating pathways involved in cell death, proliferation, inflammation, and fibrosis, which contribute to pancreatic β cell dysfunction, insulin resistance, diabetic nephropathy, and retinopathy. By analyzing current research, we aim to enhance understanding of lncRNA involvement in diabetes while identifying potential therapeutic targets and guiding future research directions to elucidate the complex mechanisms underlying this pervasive condition.

## 1. Introduction

Diabetes mellitus (DM) is a significant global public health challenge, with its prevalence continually increasing across the world. Recent estimates indicate that in 2021, approximately 537 million people were living with diabetes, with projections indicating that this number could rise to 1.31 billion by 2050 [1]. The pathophysiology of diabetes involves dysfunctions across multiple organ systems, including β islet cells, adipose tissue, skeletal muscle, and the liver. Type 1 diabetes mellitus (T1DM) results from the destruction of β cells. Type 2 diabetes mellitus (T2DM), which is more prevalent, is characterized by a complex interplay of β cell dysfunction and insulin resistance (IR). A variety of molecular, cellular, and physiological mechanisms play significant roles in the onset and progression of diabetes. Understanding the mechanisms could uncover novel therapeutic targets, potentially leading to improving treatment strategies for this pervasive condition.

Non-coding RNA (ncRNA) refers to transcripts that do not encode proteins, and accounts for over 90% of the human genomic transcripts. Among them, long non-coding RNAs (lncRNAs) represent the largest group, with lengths exceeding 200 nucleotides [2]. The latest GENCODE release (version 47) identifies a total of 50,637 lncRNA genes, including pseudogenes, resulting in 191,106 distinct lncRNA transcripts [3]. Emerging studies indicate that lncRNAs are not merely non-functional byproducts of transcription; they play crucial roles in numerous biological processes, including X chromosome inactivation, genomic imprinting, cell differentiation, apoptosis, stem cell pluripotency, nucleocytoplasmic transport, and heat shock responses [4]. Additionally, lncRNAs are implicated in various types of diseases, particularly metabolic diseases.

Diabetes is a chronic metabolic characterized by persistent hyperglycemia. Over time, this condition can cause serious damage to multiple vital organs, including the heart, blood vessels, eyes, and kidneys. Notably, stress, particularly in the form of endoplasmic reticulum (ER) stress or oxidative stress (OS), plays a key role in the development and progression of diabetic complications [5,6]. These stresses can result in outcomes like apoptosis, autophagy, cellular dysfunction, and inflammation. Research demonstrates that lncRNAs mediate the effects of ER stress and OS, influencing pathways that drive cell survival or death [7]. Existing reviews on long non-coding RNAs (lncRNAs) and diabetes have broadly explored their roles in diabetes and diabetes-related metabolic diseases but often lack a detailed examination of stress-related mechanisms [7,8,9,10]. While some reviews have addressed the pathophysiology of diabetic complications, their coverage of lncRNAs remains limited and generalized. In contrast, this review focuses specifically on stress-responsive lncRNAs, highlighting their critical roles in hyperglycemia-induced OS and ER stress, two major drivers of diabetes progression and complications. By thoroughly analyzing how these lncRNAs regulate key cellular pathways, we provided a comprehensive mechanistic perspective that bridges molecular biology with disease pathology. Furthermore, we integrated the most recent research findings on lncRNA function, identifying potential therapeutic targets and outlining promising directions for future research areas frequently underrepresented in previous reviews. We hope that our review can not only deepen the current understanding of lncRNA function in diabetes but can also offer new directions for future research and clinical applications.

## 2. Classification and Functional Mechanisms of LncRNAs

**Figure 1:**
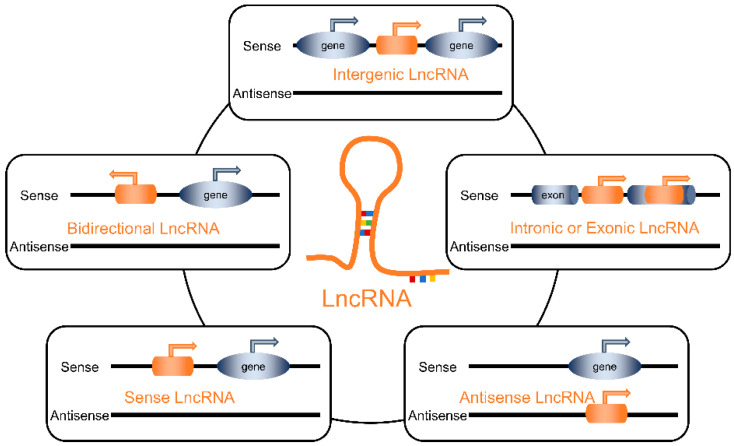
The classification of lncRNAs.

Most lncRNAs are linear, although some form circular RNAs (circRNAs) characterized by covalently linked 3’and 5’ends. Notably, the evolutionary conservation of lncRNA sequences across various species is generally low. For instance, in human cells, the majority of lncRNAs lack corresponding homologous genes in model organisms [11]. LncRNAs can be classified into five types based on their genomic positions: (1) intergenic lncRNAs: transcribed between two protein-coding genes. (2) Intronic or extronic lncRNAs: derived from the intron of a protein-coding gene. (3) Sense lncRNAs: transcribed from the sense strand of a protein-coding gene. (4) Antisense lncRNAs: transcribed from the antisense strand of a protein-coding gene. (5) Bidirectional lncRNAs: transcribed in the opposite direction from a neighboring protein-coding gene, with a transcription start site within 1 kb [12]. The classification of lncRNAs is shown in Figure 1.

In addition to their structure and classification, the characteristics of lncRNAs are also reflected in their transcription and degradation features. Although lncRNAs are primarily transcribed by RNA polymerase II and undergo mRNA-like processing, their abundance is generally low, compared with protein-coding genes and microRNAs (miRNAs) [13]. Based on the analysis of ENCODE transcripts, it has been found that except for a few lncRNAs derived from pseudogenes whose expression levels in cells are close to those of protein-coding genes, the abundance of most lncRNAs is only about one-tenth that of mRNAs, and some have only a few copies in a single cell [14]. LncRNA degradation pathways include nuclear RNA exosome-mediated degradation [15], nonsense-mediated decay [16], and conventional pathways for mRNAs, such as degradation mediated by processing bodies (p-bodies).

Studies have indicated that both lncRNAs and circRNAs play important roles in regulating biological processes by interacting with DNA, RNA, and proteins [13]. Specifically, lncRNAs can guide proteins to specific chromosomal regions or mRNAs. For example, lncHDAC2 and HAND2-AS1 can bind to chromatin remodeling complexes, thereby recruiting them to the promoters and modulating gene expression [17,18]. In the cytoplasm, the lncRNA MEG3 directs PTBP1 binding to SHP mRNA, which subsequently leads to the degradation of SHP mRNA [19]. Additionally, lncRNAs can serve as scaffolds to facilitate protein–protein interactions. HOTAIR, for instance, mediates the interactions between the SUZ12 subunit of the PRC2 transcriptional repression complex and the E3 ligase Mex3b. This interaction promotes the degradation of SUZ12 and reduces the inhibitory effects of PRC2 on target genes [20]. It is also worth noting that many lncRNAs, including circRNAs, possess miRNA binding sites and function as competing endogenous RNAs (ceRNAs). These ceRNAs can alleviate the repressive effects of miRNAs on their target genes. Notable examples include Gomafu [21], lncRNA-OGRU [22], and MIAT [23], which can, respectively, sponge miR-139-5p, miR-320, and miR-130b-3p, thereby relieving the repression exerted by these microRNAs on the expression of protein-coding genes.

It is important to emphasize that a single lncRNA may exert multiple functions. For instance, *HULC* (highly upregulated in liver cancer) acts as a scaffold for the assembly of YB-1/ERK complex [24] and a decoy to sponge miR-372/miR-373 [25], reflecting the complexity of their roles in cellular processes. Given their multifaceted roles in gene regulation and cellular mechanisms, lncRNAs represent a critical area of study for understanding metabolic disease pathology and developing therapeutic strategies.

## 3. Stresses and Stress-Associated LncRNAs

### 3.1. Endoplasmic Reticulum Stress and Major Cellular Signaling Pathways

ER stress functions as a self-protective mechanism for cells, occurring when the protein-folding capacity of the ER is overwhelmed, often due to the accumulation of unfolded or misfolded proteins. This activates the unfolded protein response (UPR), a cascade designed to restore ER homeostasis [26]. The UPR consists of three major pathways, all of which are triggered by the interaction of the chaperone protein BiP with misfolded proteins. The PERK/eIF2α pathway is initiated when misfolded proteins bind to BiP, causing its dissociation from PERK. This leads to PERK dimerization, autophosphorylation, and subsequent activation. Once phosphorylated, PERK phosphorylates eIF2α, resulting in a reduction of global protein synthesis. In the IRE1/XBP1 pathway, misfolded proteins activate IRE1, which splices XBP1 mRNA to generate XBP1s. XBP1s then upregulates genes involved in protein folding, ER-associated degradation (ERAD), and overall UPR activity, thereby enhancing cellular capacity for protein processing and quality control. Lastly, the ATF6 pathway is characterized by the translocation of ATF6 from the ER to the Golgi apparatus under ER stress. There, ATF6 undergoes proteolytic cleavage, producing an active form that upregulates essential genes involved in the unfolded protein response (UPR), including those encoding chaperones and ER-associated degradation (ERAD) components [5]. Through the orchestration of these pathways, cells adeptly manage the accumulation of misfolded proteins, thus safeguarding their functionality and ensuring cellular resilience in the face of stress.

### 3.2. Oxidative Stress and Major Cellular Signaling Pathways

OS arises from an imbalance between reactive oxygen species (ROS) production and the body’s detoxification processes. Elevated OS can damage cellular components, including lipids, proteins, and DNA, and is associated with metabolic diseases by promoting inflammation, insulin resistance (IR), and impaired metabolic function [27]. Hypoxia, characterized by deficient oxygen supply to tissues, exacerbates OS through multiple mechanisms, including mitochondrial dysfunction and disruption of antioxidant defenses [28]. Specifically, reduced oxygen availability hinders electron flow through the electron transport chain (ETC), leading to electron leakage and ROS formation, primarily from Complex I and Complex III of the ETC. Additionally, hypoxia activates the transcription factor HIF-1, which regulates genes that contribute to OS, including NADPH oxidase, an enzyme complex that generates ROS. During hypoxia, antioxidant systems are often disrupted. Initial increases in superoxide dismutase activity may occur in response to elevated ROS; however, persistent hypoxic stress can overwhelm antioxidant defenses and reduce glutathione availability, compromising the cell’s capacity to manage ROS.

### 3.3. Stresses in Diabetes and Diabetic Complications

The UPR activated by ER stress initially aims to restore ER homeostasis. However, persistent ER stress can lead to β cell death, significantly reducing the functional β cell population necessary for insulin secretion, an issue highly relevant in the context of diabetes. In an Akita mouse model, a mutation in the Ins2 gene causes proinsulin misfolding and accumulation in the ER and triggers ER stress, which, in turn, leads to pancreatic β cell death and insufficient insulin secretion, and ultimately the development of diabetes, underlining the importance of the ER in both physiologic and pathologic insulin metabolism in β cells [29]. In renal cells, ER stress may disrupt normal lipid and protein metabolism, contributing to diabetic nephropathy (DN) through mechanisms like glomerular basement membrane thickening and albuminuria development [30].

Diabetes is also associated with elevated OS. High blood glucose levels can stimulate ROS production through multiple pathways [31]. A notable pathway is the polyol pathway, where excess glucose is converted into sorbitol by aldose reductase. This reaction depletes NADPH, a vital cofactor needed for antioxidant regeneration [32]. Moreover, hyperglycemia enhances superoxide anion production in mitochondria, primarily due to increased electron leakage. The ROS generated as a result of hyperglycemia is believed to activate various stress-responsive signaling pathways, including JNK/SAPK, NF-κB, and p-38 MAPK, culminating in damage to cellular lipids, proteins, and DNA, and is implicated in the development of IR. Within the context of metabolic syndrome, elevated ROS levels impair β cell function, thereby disrupting glucose metabolism and increasing diabetes risk. Furthermore, ROSs are known to exacerbate the risk of diabetic complications. In the vascular endothelium, ROSs can oxidize low-density lipoprotein (LDL), a precursor to atherosclerosis. Likewise, OS can damage retinal cells, contributing to diabetic retinopathy (DR) [31]. Additionally, OS can also activate various pro-inflammatory signaling pathways, such as NF-κB, which further exacerbates the inflammatory response and tissue damage in diabetes [33].

Collectively, both ER stress and OS are pivotal in diabetes pathogenesis and its complications. ER stress, OS-related lncRNAs, and their roles in diabetes are presented in Table 1. Understanding these stress responses is essential for developing novel therapeutic strategies to treat diabetes and prevent its associated complications.

## 4. Stress-Associated LncRNAs Implicated in Diabetes

Emerging evidence suggests that lncRNAs play crucial roles in the development of DM and its associated complications. These molecules orchestrate various biological processes including cell death, proliferation, inflammation, and fibrosis, which are intricately interwoven with ER stress and OS. Notably, lncRNAs and stress may regulate bidirectionally, collaboratively influencing the progression of diabetes. Table 2 provides a comprehensive overview of stress-associated lncRNAs implicated in DM and diabetic complications.

### 4.1. LncRNAs Implicated in Diabetes via Regulation of Oxidative Stress

It has been demonstrated that lncRNAs influence diabetes-related conditions via OS regulation. For instance, the upregulation of ANRIL, SNHG14, and ZFAS1 contributes to DN. ANRIL promotes OS, inflammation, and cell apoptosis, leading to DN. Consistently, knocking down ANRIL in podocytes or myocardial cells can protect them from high glucose (HG)-induced OS and damage [34,44]. Similarly, silencing SNHG14 alleviates HG-induced ROS production and apoptosis, thereby reducing renal tubular injury [41]. Additionally, the downregulation of ZFAS1 exerts a protective effect against HG-induced proliferation, OS, fibrosis, and inflammation in human glomerular mesangial cells (HGMCs) [43]. In the context of diabetic cataracts (DCs), the overexpression of MALAT1 promotes apoptosis and OS through the p38MAPK pathway [47].

On the other hand, some lncRNAs can play a protective role in the development of DN. CTBP1-AS2 alleviates HG-induced OS by upregulating FOXO1, which, in turn, decreases the proliferation of HGMCs [35]. The overexpression of GAS5 inhibits inflammation, OS, and pyroptosis in renal tubular cells by downregulating the expression of miR-452-5p [45].

### 4.2. LncRNAs Implicated in Diabetes via Regulation of ER Stress

During the progression of DM and its associated complications, lncRNAs have emerged as regulators of gene expression related to ER stress. In DN, the levels of TUG1 (taurine upregulated gene 1) and LINC01619 are decreased. The overexpressing TUG1 can prevent HG-induced apoptosis and alleviate ER stress in renal epithelial cells through the miR-29c-3p/SIRT1 and PU.1/RTN1 pathways [42,49]. In contrast, the downregulation of LINC01619 triggers ER stress and podocyte injury via miR-27a/FOXO1 axis [39]. Additionally, both GAS5 and H19 are downregulated in DR or retinal cells exposed to HG, mimicking the hyperglycemic environment observed in DR, and have been shown to play a regulated role in the expression of genes associated with ER stress [37,38].

Given that lncRNAs play a substantial role in modulating diabetes progression through ER stress, it is essential to recognize that ER stress, in turn, can regulate lncRNA expression. Under diabetic conditions in renal glomeruli and mesangial cells, the lncRNA lncMGC, which contains nearly 40 microRNAs and their host long non-coding RNA transcript, is upregulated by the ER stress-related transcription factor CHOP [40]. A recent study by the same research group found that suppressing lncMGC in several types of diabetic mice, as well as in human and mouse islets, can improve glucose homeostasis and islet viability, at least in part, by attenuating ER stress-related mechanisms [48]. These findings further underscore the intricate relationship between ER stress, lncRNAs, and the pathophysiology of diabetes, highlighting potential therapeutic targets for managing this complex disease.

## 5. The Role of LncRNAs in Diabetes and Diabetic Complications

### 5.1. LncRNAs Related to Pancreatic β Cell Function

Deficiency or dysfunction of β cells is a hallmark of both T1DM and T2DM, although the underlying mechanisms diverge significantly. In T1DM, the immune system mistakenly attacks and destroys insulin-producing β cells, resulting in their loss and subsequently hyperglycemia due to insufficient insulin production [50]. Additionally, pancreatic β cells express low levels of antioxidant enzymes, rendering them particularly susceptible to OS and ER stress, which can further contribute to β cell death [51,52]. In contrast, T2DM initially manifests as IR, where the body’s cells inadequately respond to insulin. As T2DM advances, β cells are unable to synthesize adequate insulin to satisfy the elevated demand, leading to dysfunction. Ultimately, both conditions result in hyperglycemia and associated complications.

The contribution of lncRNAs to pancreatic islet physiology and disorders was first elucidated by Ding and colleagues, who demonstrated that lncRNA H19 is implicated in the dysfunction of islets resulting from gestational diabetes [53]. In a subsequent investigation, Fadista and collaborators established a positive association between the expression of lncRNA LOC283177 with insulin exocytosis and key genes critical for normal islet function, such as Pax6, Syt11, and Madd [54]. Arnes and his team identified lncRNA β cell long intergenic noncoding RNA 1 (βlinc1) as a cis-regulator of the islet transcription factor NKX2.2, showing that βlinc1 knockout mice exhibit impaired glucose tolerance due to defects in islet development [55]. In addition, transcriptome profiling studies of islets and β cells have identified over 1000 islet-specific lncRNAs in both human and mouse islets. Notably, a subset of these lncRNAs is mapped to established susceptibility loci for T2DM pathogenesis, suggesting their roles in diabetes. KCNQ1OT1, an antisense lncRNA that is upregulated in islets from patients with T2DM [56], has been linked to T2DM through genome-wide association studies (GWASs) [57]. Additionally, Akerman’s group demonstrated that concomitant downregulation of lncRNA PLUTO and PDX1 (encoding a key β cell transcription factor) can be observed in islets from donors with T2DM or impaired glucose tolerance, and PLUTO knockdown reduced the contact between upstream enhancers and the *PDX1* promoter [58], suggesting that PLUTO affects local 3D chromatin structure and consequently transcription of PDX1.

LncRNA β cell function and apoptosis regulator (βFaar) is downregulated in islets from obese mice, contributing to β cell dysfunction and apoptosis. Both in vitro and in vivo functional assays have demonstrated that βFaar overexpression enhances insulin production to protect β cell from apoptosis, and upregulates Ins2, NeuroD1, and Creb1 expression by sponging miR-138-5p. Additionally, βFaar also stabilizes TRAF3IP2 by preventing its degradation, thereby inhibiting NF-κB-mediated apoptosis [59]. The ER stress-regulated lncMGC is important in pancreatic islets and T1DM pathology. In vivo, lncMGC-KO mice treated with streptozotocin (STZ) had lower blood glucose levels (BGLs) and HbA1c than wild types. GapmeR targeting lncMGC reduced insulitis and BGLs in T1DM non-obese diabetic mice, and also in T1DM Akita mice compared to controls. Additionally, lncMGC-GapmeR enhanced islet viability from human donors and lncMGC mice ex vivo. These findings show that GapmeR targeting lncMGCs can effectively alleviate diabetes in mice and preserve islet viability, highlighting its therapeutic potential [48].

LncRNAs that regulate β cell proliferation and differentiation also affect islet function. TCL1 upstream neural differentiation-associated RNA (TUNAR) was enriched in islet and was upregulated in β cells in individuals with T2DM. In EndoC-βH1 cells, silencing TUNAR inhibited the 1-AKP-induced Wnt/β-catenin signaling pathway and cell proliferation through elevation of DKK3, a known Wnt antagonist, without affecting insulin secretion or β cell apoptosis [60]. Interestingly, Akerman et al. revealed that lentiviral shRNA-mediated silencing of TUNAR (referred to as HI-LNC78) resulted in decreased insulin content and impaired glucose-stimulated insulin secretion in T-antigen-excised EndoC-βH3 cells [58]. Given these discrepancies, both the results and underlying mechanisms demand further investigation. A comprehensive single-cell RNA-sequencing analysis of the various differentiation stages from the hESC-H9 line to β cells identified MIR503HG as a highly expressed lncRNA crucial for pancreatic progenitor development. The knockout of MIR503HG was found to promote the differentiation of pancreatic progenitors and enhance insulin synthesis and secretion in mature β cells derived from stem cells in vitro. Furthermore, transplantation of MIR503HG KO stem-cell-derived β cells into diabetic mice restored blood glucose homeostasis. Mechanistically, MIR503HG regulated pancreatic progenitor differentiation by directly interacting with CtBP1 and relieving the transcriptional corepression effect exerted by CtBP on E-cadherin and HES1 expression [61].

Interestingly, multiple genome-wide association studies have shown that the majority of single-nucleotide polymorphisms (SNPs) associated with the risk of T2DM risk are located within non-coding regions of the human genome [62,63]. One such lncRNA, Reg1cp, is mainly expressed in islets. An association study conducted with 16,113 Chinese adults identified that the rs3819316 C > T mutation in Reg1cp (referred as Mut-Reg1cp) increases T2DM risk. Mice with a β cell-specific knock-in of Mut-Reg1cp exhibited impaired glucose metabolism and β cell dysfunction. This effect is attributed to Mut-Reg1cp directly binding to polypyrimidine tract-binding protein 1 (PTBP1), which inhibits its phosphorylation, ultimately reducing insulin mRNA stabilization and secretion. Furthermore, exosomes derived from islets can transfer Mut-Reg1cp into peripheral tissues, including the liver and muscle. Once there, Mut-Reg1cp promotes IR by suppressing the translation of AdipoR1 and disrupting adiponectin signaling [64].

### 5.2. LncRNAs Related to Insulin Resistance

Insulin is secreted by pancreatic β cells, whereas insulin receptors are present on insulin target cells such as skeletal muscle cells, hepatocytes, and adipocytes [65]. IR is a key risk factor and a major T2DM characteristic, characterized by reduced peripheral glucose uptake, increased liver glucose production, and impaired insulin secretion and insulin sensitivity. Several factors contribute to IR, including high levels of free fatty acids associated with obesity, as well as hyperglycemia and pro-inflammatory cytokines such as TNF-α, which further exacerbate metabolic dysfunction.

LncRNAs play significant roles in the regulation of IR through their expression in various tissues, mainly insulin-target tissues. In adipose tissue, two adipocyte-specific metabolic-related (ASMER) lncRNAs, ASMER-1 and ASMER-2, have been identified to be upregulated in white adipose tissue (WAT) of obese insulin-resistant women compared to those who were insulin-sensitive. Knockdown of the in vitro differentiated adipose-derived stromal cells decreased glycerol release and secretion of adipokines like adiponectin, although they did not seem to affect insulin-induced lipogenesis directly. The mechanisms involve complex interactions with essential regulatory genes, such as PPARG and INSR, suggesting their crucial involvement in maintaining adipocyte function [66]. Similarly, lncRNA ADIPINT expression is increased in the adipose tissue of obese humans and downregulated in WAT following gastric bypass surgery. Its expression was associated with fat cell size and adipose IR. Knocking down ADIPINT in vitro significantly reduced pyruvate carboxylase activity and consequently impaired glycerol release and insulin-stimulated lipid synthesis. This highlights its pivotal role in overall adipocyte metabolism and IR [67]. In addition, Lu et al. revealed that lncRNA MGE3 knockdown reduced TNFα-indued ROS generation and cell apoptosis of adipocyte in vitro and high-fat diet (HFD)-induced chronic inflammation and IR in vivo [68]. Consistently, analysis with clinical samples showed that MEG3 expression in adipose tissues from obese women was positively associated with IR indices [69].

In the liver, several lncRNAs contribute to insulin sensitivity, including MEG3, MALAT1, and Gomafu. MEG3 expression rises in palmitate-treated primary hepatocytes and liver tissue of in HFD or ob/ob mice [70,71], and has been shown to impair glycogen accumulation and promote IR by serving as a ceRNA of miR-214 to facilitate ATF4 expression [72]. Evidence also suggests that lowering MEG3 expression by intravenous tail vein injection of MEG3 siRNA can alleviate hepatic fat accumulation and enhance glucose tolerance in HFD mice and ob/ob mice [71]. Gomafu, an lncRNA with increased expression in the livers of obese mice, has also been implicated in promoting glucose tolerance and IR by acting as a sponge of miR-139, leading to the de-repression of its target gene, FOXO1, and, consequently, increases glucose production in hepatocyte. Importantly, the knockdown of hepatic Gomafu via admiration of lipid nanoparticle-formulated siGomafu in obese mice improved insulin sensitivity and decreased hepatic glucose production in obese mice [21].

Although MALAT1 is not essential for development in a knockout mouse model under normal physiological conditions [73,74,75], MALAT1 has emerged as a player in IR-related metabolic disorders, especially in the liver. MALAT1 is upregulated in both liver cells exposed to saturated fatty acids and in the livers of obese mice. Reducing MALAT1 levels can improve insulin sensitivity and protect against OS, indicating its regulatory role in the liver. Notably, tail-vein-injected lipid nanoparticle-formulated siMALAT1 improves insulin sensitivity in ob/ob mice [76]. Similarly, MALAT1-null mice showed sensitized responses to insulin. Ex vivo studies on MALAT1-null hepatocytes and islet cells showed that MALAT1 ablation sensitized insulin responses and was protected against OS [77].

Additionally, the lncRNA suppressor of hepatic gluconeogenesis and lipogenesis (lncSHGL) and its human homologous lncRNA B4GALT1-AS1 were downregulated in the livers of obese mice and patients with nonalcoholic fatty liver disease. These lncRNAs suppress gluconeogenesis and lipogenesis through their interaction with heterogeneous nuclear ribonucleoprotein A1 (hnRNPA1), which leads to enhanced translation of calmodulin (CaM) protein and improved metabolic profiles. Specifically, this interaction activates the CaM/Akt pathway and represses the mTOR/SREBP-1C pathway in models of obesity and diabetes. Restoration of hepatic lncSHGL has been found to improve hyperglycemia, insulin resistance (IR), and steatosis in obese diabetic mice, suggesting that activating the lncSHGL/hnRNPA1 axis could be a potential strategy for the treatment of T2DM and steatosis [78].

In skeletal muscle, lncRNA H19 exhibits reduced expression in T2DM patients and insulin-resistant rodent models [79,80]. Overexpression of H19 in db/db mice can reverse glucose intolerance and boost insulin sensitivity by mitigating ectopic lipid deposition in skeletal muscle and promoting fatty-acid-oxidation-related gene translation associated with fatty acid oxidation [79]. Furthermore, its interaction with let-7 forms a double-negative feedback loop, regulating insulin sensitivity [80]. Additionally, using a whole-body H19 KO mouse model, Geng et al. established that the deletion of H19 leads to muscle IR via inhibition of AMPK/PGC-1α signaling. Downregulation of H19 in myotubes decreased DUSP27 phosphatase expression at both the mRNA and protein levels, and DUSP27, in turn, interacts with AMPK to enhance AMPK activity [81]. Collectively, these findings underscore the pivotal role of H19 in the regulation of insulin sensitivity and lipid metabolism within skeletal muscle.

Another lncRNA, EPB41L4A-AS1, was abnormally increased in the liver of patients with T2DM and the muscle cells of patients with IR and T2DM. It affects glucose uptake and mitochondrial respiration by enhancing GLUT2/4 endocytosis and inhibiting GLUT4 transcription by interacting with GCN5 and modulating histone modifications. In vivo, expressing human EPB41L4A-AS1 in mice increased both fasting blood glucose levels and HG-charged blood glucose levels, indicating impaired glucose tolerance [82].

lncRNA TUG1 was downregulated in the islet of HFD-induced gestational diabetes mellitus. Restoration of lncRNA TUG1 by tail vein injections of nanoparticles expressing lncRNA TUG1 alleviative IR in GDM mice, as evidenced by the reduced fasting blood glucose, fasting insulin, homeostasis model assessment of IR, HOMA pancreatic β cell function, insulin sensitivity index for oral glucose tolerance tests, and insulinogenic index levels in mouse serum [83]. For a more comprehensive summary of lncRNA associated with IR, please refer to Table 3.

### 5.3. LncRNAs Related to Diabetic Complications

It is well-established that severe hyperglycemia plays a central role in the pathogenesis of both microvascular and macrovascular complications, including DN, DR, and DCM [86]. Recent research highlights the significant role of OS, resulting from the excessive production of ROS induced by hyperglycemia, in the onset and progression of these diabetic complications. This hyperglycemia-driven ROS generation is thought to activate various stress-responsive signaling pathways, further exacerbating the complications associated with T2DM. Additionally, a complex interplay of genetic and environmental factors, including obesity, dyslipidemia, and hypertension, accelerates the progression of T2DM-related complications, adding to the overall complexity of the disease.

#### 5.3.1. LncRNAs Associated with DN

DN represents a microvascular complication of diabetes and the leading cause of end-stage renal disease in most developed countries. Its features include excessive albuminuria, glomerular matrix accumulation, glomerular hypertrophy, and renal fibrosis. Targeting early features of DN, including renal extracellular matrix (ECM) accumulation and glomerular hypertrophy, may effectively prevent disease progression [87].

In the context of DN, various renal cell types are predominantly affected. Mesangial cells, in particular, play a critical role, characterized by aberrant proliferation and overproduction of ECM, which contributes to glomerular matrix accumulation. Furthermore, podocytes, which are vital for maintaining the integrity of the glomerular filtration barrier, experience substantial damage. This injury compromises their function, resulting in elevated albuminuria due to the impaired barrier. Additionally, renal proximal tubule epithelial cells are implicated in this pathology; they are involved in the progress of renal fibrosis, which exacerbates the decline of kidney function. Collectively, the dysregulation of these cell types is fundamental to the development and progression of DN.

Research has explored the roles of multiple lncRNAs in DN. First, lncRNA TUG1 is downregulated in diabetic mice podocytes and human DN subjects as well as in HG-treated kidney cell lines. TUG1 protects podocytes from HG-induced mitochondrial ROS and cell death. Podocyte-specific TUG1 overexpression in diabetic mice reduces albuminuria and mesangial matrix expansion and improves podocyte foot process effacement and glomerular basement membrane thickening [88]. However, the renoprotective effects of overexpressing TUG1 in podocytes are lost when *Pgc1α* is knocked out, suggesting that PGC1α is required for the renoprotective effect of TUG1 in vivo. Mechanistically, HG represses TUG1 transcription via transcription factor carbohydrate response element binding protein (ChREBP) and its partners [89]. TUG1 interacts with PGC-1α, promoting PGC-1α binding to its promoter and enhancing mitochondrial function, such as increased mitochondrial biogenesis and reduced ROS, partially dependent on arginase 2 activity [90]. Interestingly, downregulation of TUG1 was also detected in renal tubular epithelial cells of DN mice and HG-treated human renal tubular epithelial cell lines. It was found that TUG1 reduced HG-induced ER stress and apoptosis in vitro. In vivo experiments also verified that TUG1 inhibited ER stress and apoptosis and alleviated renal tubular lesions in DN mice [49]. The lncRNA MIAT is upregulated patients’ plasma and kidney tissues in DN. It promotes podocyte injury and mitotic catastrophe via the miR-130b-3p/Sox4/p53 pathway. Depleting MIAT or inhibiting miR-130b-3p can alleviate podocyte damage, suggesting MIAT as a potential therapeutic target [23].

MALAT1 levels were increased in kidney cortices from C57BL/6 mice with STZ-induced DN and in mouse podocytes under short-time HG treatment. And knockdown of MALAT1 inhibits HG-induced EMT of podocytes, as evidenced by increased p-cadherin and ZO-1 and decreased desmin expression and MMP-2 activity [91]. lncRNA PVT1 is a nucleus-located lncRNA; lncRNA PVT1 significantly increased in the serum of DN patients and HG-induced-treated primary podocytes or immortalized mouse podocyte cell line compared with their counterparts. PVT1 promotes the H3K27me3 of the FOXA1 promoter by recruiting EZH2 and thus inhibits the expression of FOXA1 to promote the apoptosis and damage of podocytes in DN. Silencing of PVT1 inhibited the apoptosis and damage of podocytes in DN in vivo. PVT1 inhibits anti-apoptotic Bcl-2 expression and promotes Bax expression [92]. Similarly, Zhong et al. showed that lncRNA PVT1 is also upregulated in HG-induced-treated primary mesangial cells, and knockdown of lncRNA PVT1 inhibits HG-induced proliferation and the expression of fibrosis-related proteins α-SMA and fibronectin in human renal mesangial cells by functioning as a ceRNA of miR-23b-3p [93].

LncMGC is upregulated in mesangial cells exposed to TGF-β1 or HG conditions, both of which are associated with DN. Targeting lncMGC with a chemically modified oligonucleotide has been shown to reduce glomerular ECM accumulation and hypertrophy in diabetic mice, highlighting its potential as a therapeutic target for controlling DN progression [40]. Additionally, the knockdown of Gm4419 decreases pro-inflammatory cytokine expression and renal fibrosis markers, and inhibits cell proliferation in mesangial cells under HG conditions. In contrast, overexpression of Gm4419 promotes inflammation, fibrosis, and proliferation in mesangial cells in low-glucose conditions. Notably, Gm4419 activates the NF-κB pathway by directly interacting with p50, a subunit of NF-κB, and interacts with the NLRP3 inflammasome in mesangial cells, reinforcing its role in the inflammation and progression of DN [94].

NEAT1 l is upregulated in the kidneys of HFD- and STZ-induced diabetic mice, rats, and HG-treated mouse mesangial cells or bovine serum albumin (BSA)-treated tubular epithelial cells [95,96,97,98]. Gain- and loss-of-function studies revealed that NEAT1 promoted BSA-induced expression of fibrotic and EMT-related genes via the ERK1/2 pathway [95]. Knockdown of NEAT1 inhibited proliferation and repressed ECM protein secretion in mesangial cells in vitro through activation of the Akt/mTOR signaling pathway [96] or by targeting miR-27b-3p and ZEB1 [97] or the miR-222-3p/CDKN1B axis [98], and inhibited STZ-induced renal damage in diabetic rodents in vivo.

In the kidney tissues of patients with DN and human renal tubule epithelial cell lines treated with HG, the levels of ANRIL and thioredoxin-interacting protein (TXNIP) were increased. Investigations based on in vitro cell lines demonstrated that ANRIL facilitates cell pyroptosis, by functioning as a sponge for miR-497. This action relieves the inhibition of TXNIP, which serves as a marker for an excessive UPR [99]. In the STZ-induced diabetes mouse model, ANRIL knockout reduced expressions of ECM proteins and decreased urine albumin levels in comparison with the wild-type diabetic animals, suggesting a protective effect on diabetic mouse kidneys [100].

KIFAP3-5:1 is downregulated in plasma samples, db/db mouse kidney tissues, and HG-treated renal tubular epithelial cells. It directly targets the −488 to −609 element in the promoter of the paired related homeobox 1 (PRRX1), an inducer of EMT, suppresses PRRX1 expression, and ameliorates EMT in renal tubular epithelial cells under HG conditions. Administration of lentivirus expressing KIFAP3-5:1 through tail vein injection ameliorates renal function and renal interstitial fibrosis in db/db mice, as indicated by reduced blood urea nitrogen levels, and reduced the levels of renal tubular injury markers such as NAG, urinary protein, and β2-MG [101].

**Figure 2:**
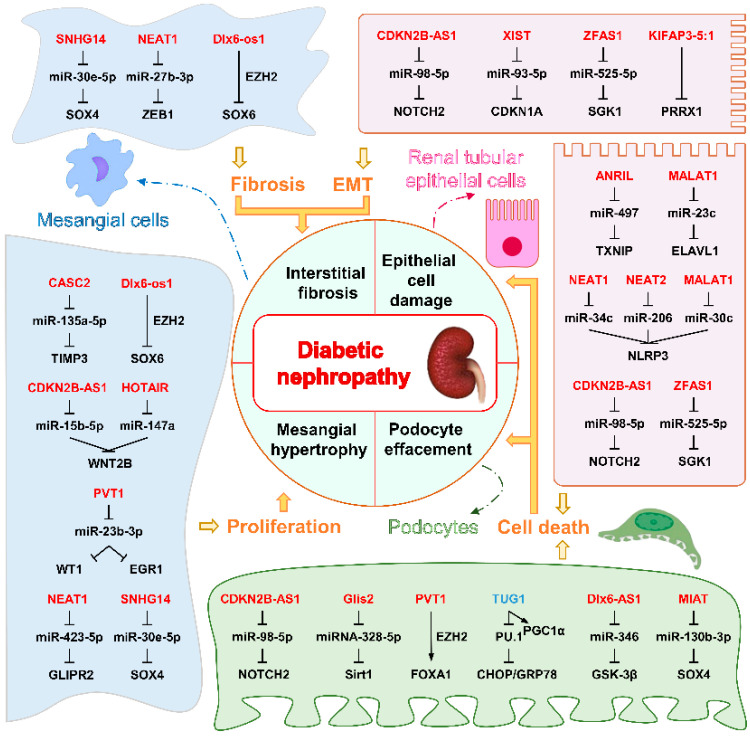
The role of lncRNAs in DN. The pathophysiology of DN includes interstitial fibrosis, mesangial hypertrophy, epithelial cell damage, and podocyte effacement. LncRNAs modulate processes such as epithelial–mesenchymal transition (EMT), fibrosis, proliferation, and cell death, ultimately contributing to the pathological manifestations of DN. Under the condition of DN, the downregulated expression of lncRNAs is marked in blue, and the upregulated expression of lncRNAs is marked in red. Abbreviations are specified in the Abbreviations section.

Similar to the observation podocytes [91], MALAT1 was also upregulated in the HG-treated tubular epithelial cell line [102,103]; cell-based loss of function analyses revealed that knockdown of MALAT1 in suppressed HG-induced EMT and tubular epithelial cell death [102,103], although the underlying mechanisms remains to be determined. Consistently, overexpression of MALAT1 enhanced HG-induced ROS generation, secretion of inflammatory cytokines, and cell apoptosis, and in vivo knockdown of MALAT1 through intravenous injection of a lentivirus expressing sh-MALAT1 alleviated renal tubular epithelial injury by suppressing LIN28A and the Nox4/AMPK/TOR signaling in rats with DN [104]. A summary of the role of lncRNAs in DN is shown in Figure 2.

#### 5.3.2. LncRNAs Associated with DR

DR is characterized by a progressive transformation of the retinal microvasculature, encompassing increased retinal nonperfusion, enhanced vasopermeability, and pathological proliferation of retinal vessels. A growing body of research has identified several lncRNAs that exhibit altered expression patterns in both the retinal tissue of DR patients and in mouse models, as well as in retinal cells exposed to HG, a condition that mirrors the hyperglycemic environment, seen in diabetic retinopathy.

Notably, several lncRNAs are significantly downregulated in the retinal tissues of both patients and mouse models under HG conditions. For instance, SNHG7 is decreased in retinal endothelial cells exposed to HG, leading to increased cell proliferation and apoptosis by sponging miR-34a-5p and promoting VEGF expression, thereby contributing to abnormal angiogenesis [105]. Similarly, lncCytB, encoded by mitochondrial DNA, shows reduced expression in retinal microvessels under diabetic conditions [106,107]. Its downregulation compromises the activity of the cytochrome B complex III, resulting in increased ROS production, a key factor driving mitochondrial dysfunction and DR progression [108].

VEAL2 is also downregulated in retinal choroidal tissues of DR patients; its loss due to elevated diacylglycerol levels in endothelial cells promotes junctional protein degradation, resulting in enhanced hyperpermeability and vascular leakage [109]. Collectively, the downregulation of these lncRNAs under hyperglycemic conditions leads to increased OS, impaired endothelial function, and progression of diabetic retinopathy through various mechanisms including ROS production and disruption of endothelial integrity.

Conversely, MALAT1 is upregulated in the retinal tissue of diabetic patients and mouse models, as well as in retinal endothelial cells under HG [110]. Knockdown of MALAT1 has been shown to ameliorate apoptosis in retinal cells from STZ-induced diabetic rats and enhance retinal vessel function by inhibiting pericyte loss, capillary degeneration, microvascular leakage, and retinal inflammation [111]. In cultured endothelial cells, overexpression of MALAT1 has been found to increase Nrf2 protein levels and inhibit H_2_O_2_-induced OS, lipid peroxidation, and DNA damage [112], suggesting a protective effect for MALAT1 in endothelial cells.

Furthermore, MEG3 was found to be overexpressed in diabetic rat models through intravitreal administration of a MEG3-expressing virus. Overexpression of MEG3 was shown to suppress endothelial–mesenchymal transition (EndMT) in these models by inhibiting the PI3K/Akt/mTOR signaling pathway, thus preserving retinal structure and reducing the loss of retinal ganglion and granular cells, compared to those treated with a control virus. Notably, DNMT1 was identified as a promoter of MEG3 promoter methylation, inhibiting MEG3 expression by recruiting methyltransferases that activate the PI3K/Akt/mTOR pathway, thereby accelerating EndMT in DR. This study highlights the inhibitory effect of MEG3 on EndMT in DR, positioning it as a promising therapeutic target for treatment [113].

Another lncRNA, OGRU, was found to be upregulated in the retinal tissue of patients and mouse models, as well as in HG-treated Müller cells. In Müller cells, OGRU knockdown reduced VEGF and TGF-β1 expression, mitigated inflammation and OS by regulating related signaling pathways. It acts as a ceRNA for miR-320 to regulate USP14, which subsequently enhances TGF-β1 signaling and ultimately contributes to DR progression [22]. Moreover, Müller-cell-derived exosomes delivered OGRU to microglia, promoting their polarization toward the M1 phenotype. Mechanistically, OGRU served as a ceRNA for miR-320-3p, miR-221-3p, and miR-574-5p, regulating the expression of aldose reductase (AR), PFKFB3, and glucose transporter 1 (GLUT1) in microglia, respectively. The loss of miR-320-3p, miR-221-3p, or miR-574-5p or overexpression of AR, PFKFB3, or GLUT1 abolished the effect of OGRU silencing on microglia polarization in vitro. In vivo studies further demonstrated that the OGRU/miR-320-3p/AR, OGRU/miR-221-3p/PFKFB3, and OGRU/miR-574-5p/GLUT1 axes regulate microglia polarization in DR mice. Collectively, Müller-cell-derived exosomal OGRU plays a critical role in modulating microglia polarization in DR, impacting the progression of this debilitating condition [114].

DCs are another diabetes-related ocular disease arising from hyperglycemia in diabetes. In this condition, glucose penetrates the lens, where it is enzymatically converted into sorbitol by aldose reductase. This metabolic alteration induces osmotic changes that result in lens fiber damage. Moreover, elevated glucose levels provoke OS, leading to the oxidation and aggregation of proteins within lens cells, which further contributes to cataract formation. LncRNAs MALAT1 and PVT1 emerged as contributors to the pathogenesis of DC development. Gong et al. revealed that MALAT1 expression is markedly elevated in DC anterior lens capsule tissues and HG-treated lens epithelial cells. Notably, MALAT1 facilitates HG-induced OS and apoptosis in human lens epithelial cells by activating the p38MAPK signaling pathway [47]. Similarly, lncRNA PVT1 was significantly upregulated in the DC tissue and the HG-induced lens epithelial cell line. In vitro, loss of function studies showed that silencing PVT1 abrogated HG-induced apoptosis and inhibitory effect on lens epithelial cell proliferation [115]. A summary of the role of lncRNAs in DR is exhibited in Table 4.

## 6. Conclusions and Further Perspectives

In this review, we highlight lncRNAs that have been experimentally validated to modulate the key hallmarks of diabetes and diabetic complications. Many of these respond to stressors, particularly hyperglycemic conditions that mimic the hyperglycemic environment; others regulate critical pathways associated with ER stress or OS. Our growing understanding of the complex regulatory network of lncRNAs in diabetes development and progression will pave the way for lncRNA-based therapeutics.

Diabetes is a systemic disease that involves complex interactions among multiple organs and tissues. Therefore, in vivo models play a vital role in elucidating its intricate pathology, as in vitro studies lack intricate interactions with other cell types, tissues, and systemic factors that influence the pathogenesis of diabetes and cellular stress responses [116]. Of note, a substantial number of lncRNAs are uniquely present in the human genome [117]. Current research utilizing animal models primarily focuses on conserved lncRNAs, resulting in the roles of less conserved lncRNAs in diabetes and its complications remaining largely unexplored. In addition, the limited conservation across species hinders the effective translation of findings into relevant in vivo settings. Advancing humanized animal models or employing human-induced pluripotent stem-cell-based engineered tissues may help address this limitation.

Though lncRNAs show relatively poor conservation among species, they exhibit distinct expression profiles in various tissues and cell types, offering promising opportunities for precision medicine [118]. By developing therapeutics that specifically target unique non-coding RNAs in pathological cells, reduction in the impact on normal cells can be anticipated, thereby reducing adverse side effects [119]. Moreover, lncRNAs participate in complex interactions with DNA, RNA, and proteins, significantly influencing the regulation of multiple genes and signaling pathways. For instance, lncRNA MALAT1 has demonstrated simultaneous regulatory effects on diabetes and related complications, such as DN and DR [47,91,102,103,104,112]. Targeting MALAT1 presents an opportunity to concurrently address multiple pathological processes, underscoring its substantial potential in treating complex diseases like diabetes.

Diverse strategies can be employed in lncRNA-based interventions, one of which leverages nucleic acid therapeutics, such as small interfering RNA (siRNA), to directly target and inhibit lncRNAs, thus facilitating their degradation [120]. The advent of patisiran, the first siRNA drug to receive regulatory approval [121,122], has significantly reshaped the landscape of siRNA research and development, paving the way for the design of siRNA therapies specifically aimed at lncRNAs in the context of diabetes treatment. Furthermore, innovative approaches, such as the development of small molecule compounds like RIBOTACs, which promote the selective degradation of targeted RNAs [123,124], herald new possibilities for drug discovery and enhance the potential for effective therapeutic interventions.

Finally, lncRNAs show significant promise for early diagnosis and prognosis of diabetes and its associated complications. Notably, variations in the expression of lncRNAs appear in the early stages of the disease and correlate with disease progression and outcomes. In particular, those with altered levels in plasma or peripheral blood mononuclear cells may serve as valuable early diagnostic markers [23,101,125]. Furthermore, the dynamic changes in their expression may provide real-time indicators of the disease process, facilitating optimal treatment timing and enabling tailored therapeutic regimens. This capability is crucial for implementing early interventions and precise management strategies, ultimately enhancing the overall approach to diabetes care.

In conclusion, the potential of lncRNA-targeting therapies and detection strategies is becoming increasingly evident, although translating lncRNA research into clinical applications remains challenging. A deeper understanding of the functions and mechanisms of lncRNAs in diabetes aims to identify new therapeutic targets and biomarkers to combat this complex disease.

## Figures and Tables

**Table 1:** ER stress, OS-related lncRNAs, and their roles in diabetes.

LncRNAs	Mechanism in ER Stress or OS	Impact on Diabetes	Reference
ANRIL	ANRIL knockdown decreased the level of MDA.	Accelerates podocytes inflammation in HG condition	[34]
CTBP1-AS2	CTBP1-AS2 overexpression reduced ROS and MDA levels, and increased SOD activity.	Protects HGMCs from HG-induced accumulation of extracellular matrix, and inflammation	[35]
FLG-AS1	FLG-AS1 overexpression inhibited ROS, MDA levels.	Protects retinal pigment epithelial cells from HG-induced inflammation, and apoptosis	[36]
GAS5	GAS5 overexpression reduced ATF4, CHOP, as well as p-PERK/PERK and p-eIF2α/eIF2α levels.	Protects retinal epithelial cells from HG-induced apoptosis and inflammation	[37]
H19	H19 overexpression enhanced the expression of XBP1s.	Protects retinal pigment epithelial cells from HG-induced inflammation	[38]
LINC01619	LINC01619 knockdown upregulated CHOP and GRP78 levels.	Protects podocytes from HG-induced injury	[39]
MGC	CHOP upregulated lnc RNA MGC expression in glomeruli.	Accelerate mesangial cell hypertrophy and the accumulation of extracellular matrix in HG condition	[40]
SNHG14	SNHG14 knockdown inhibited ROS levels.	Accelerates renal epithelial cells apoptosis in HG condition	[41]
Tug1	TUG1 overexpression reduced GRP78, CHOP, p—PERK, and p-eIF2α levels.	Protects renal tubular epithelial cells from HG-induced apoptosis	[42]
ZFAS1	ZFAS1 knockdown inhibited ROS, MDA levels and increased SOD activity.	Accelerates mesangial cells fibrosis, and inflammation in HG condition	[43]

Note: Abbreviations are specified in the Abbreviations section.

**Table 2:** The roles and mechanisms of stress-associated lncRNAs in diabetes and its complications.

LncRNAs	Diabetic Complications	Expression Levels in Diabetes	Association to Stresses and Mechanism in Diabetes	Reference
ANRIL	DN and DM	Up	Silencing ANRIL through MME alleviates inflammation, OS, and apoptosis in podocytes while also reducing myocardial injury in diabetes by inhibiting myocardial OS.	[34,44]
CTBP1-AS2	DN	Down	CTBP1-AS2 alleviated HG-induced OS, ECM accumulation, and inflammation in HGMCs via miR-155-5p/FOXO1 axis.	[35]
FLG-AS1	DR	Down	Overexpression of FLG-AS1 reduced inflammation, OS, and apoptosis of HG-treated human retinal pigment epithelial cells via the miR-380-3p/SOCS6 axis.	[36]
GAS5	DN/DR	Down	GAS5 overexpression inhibited inflammation, OS, and pyroptosis in renal tubular cells by downregulating miR-452-5p expression, while also reducing ER stress-related apoptosis and inflammation in retinal pigment epithelium cells via SERCA2b.	[37,45]
H19	DR	Down	H19 overexpression inhibited inflammatory processes via XBP1s/miR-93 in human retinal pigment epithelial cells.	[38]
HOTAIR	DCM	Down	HOTAIR overexpression decreased OS and inflammation, and attenuated myocyte death via miR-34a/SIRT1 signaling.	[46]
LINC01619	DN	Down	LINC01619 downregulation triggered ER stress and podocyte injury via miR-27a/FOXO1.	[39]
MALAT1	DC	Up	MALAT1 overexpressed promoted apoptosis and OS via the p38MAPK pathway in human lens epithelial cells.	[47]
MGC	DN and DM	Up	The ER stress-related transcription factor CHOP upregulated MGC in glomeruli, while lowering MGC reduced cell death in pancreatic islets.	[40,48]
SNHG14	DN	Up	Silencing SNHG14 inhibited apoptosis, OS, and inflammation through the miR-483-5p/HDAC4 pathway to mitigate renal tubular damage.	[41]
TUG1	DN	Down	Overexpressed TUG1 could prevent HG-induced apoptosis and alleviate ER Stress in renal epithelial cells via miR-29c-3p/SIRT1 and PU.1/RTN1 pathway.	[42,49]
ZFAS1	DN	Up	Silencing ZFAS1 had a protective effect on HG-induced proliferation, OS, fibrosis, and inflammation in HGMCs.	[43]

Note: Up, upregulated; Down, downregulated. Abbreviations are specified in the Abbreviations section.

**Table 3:** Functional mechanisms of lncRNAs associated with IR in diabetes.

LncRNAs	Model of DM	Expression Levels in DM	Functions in DM	Target	Reference
ADIPINT	T2DM	Upregulated in abdominal subcutaneous adipose tissue with obese women	Enhances the synthesis of triglycerides and increases the size of the lipid droplet/fat cell	Pyruvate carboxylase	[67]
ASMER-1/2	T2DM	Upregulated in adipocyte tissue with obese women	Enhances lipolysis, adiponectin release, and triglyceride accumulation	PPARG and INSR	[66]
betaFaar	T2DM	Downregulated in the islets of the obese mice	Enhances β cell apoptosis to decrease insulin transcription and secretion	TRAF3IP2/NF-κB (β cell apoptosis), miR-138-5p (insulin transcription)	[59]
EPB41L4A-AS1	T2DM	Upregulated in the liver of patients with T2DM, muscle cells of patients with IR, and T2DM cell models	Inhibits glucose uptake and mitochondrial respiration in liver cells	GLUT2/4/TXNIP	[82]
Gomafu	T2DM	Upregulated in the livers of obese mice	Enhances hepatic gluconeogenesis and decreases insulin sensitivity	miR-139/FOXO1	[21]
H19	T2DM	Downregulated in skeletal muscle of humans with T2DM, HFD mice and db/db mice	Impairs systemic glucose metabolism, decreases expression of insulin receptor and lipoprotein lipase, enhances lipid deposition in skeletal muscle, impairs adipogenesis, oxidative metabolism, and mitochondrial respiration in brown adipocytes	hnRNPA1/PGC1a, miRNA let-7, DUSP27/AMPK, PEG-inactivating H19-MBD1 complexes	[79,80,81,84]
MALAT1	T2DM	Upregulated in patients with T2DM, in hepatocytes exposed to palmitate, livers of ob/ob mice, and palmitate-treated primary hepatocytes	Inhibits glucose uptake and insulin signaling response, enhances lipid accumulation in hepatocytes, impairs insulin secretion, and decreases pancreatic islet cellularity	SREBP-1c, Nrf2, miR-382-3p/resistin,	[76,77,85]
MEG3	T2DM	Upregulated in the livers of HFD and ob/ob mice, palmitate-treated primary hepatocytes, in adipocytes treated with TNF-α and HFD mice	Impairs glucose and insulin tolerance, enhances hepatic lipid accumulation, Enhances adipocyte inflammation injury	miR-185-5p/Egr2, FOXO1, miR-214/ATF4, IGF2BP2/TLR4/NF-κB	[68,70,71,72]
MIR503HG	T1DM	Not detected	SC-β cell differentiation and insulin production	CDH1, HES1	[61]
SHGL	T2DM	Downregulated in obese mouse livers	Inhibits hepatic gluconeogenesis and lipogenesis	hnRNPA1/CaM/Akt	[78]
TUG1	GDM	Downregulated in islet tissues of mice with HFD-induced GDM	Enhances β cell apoptosis	miR-328-3p/SREBP-2/ERK	[83]
TUNAR	T2DM	Downregulated in β cells of T2DM patients	Inhibits β cell proliferation	Wnt/DKK3	[60]

Note: Abbreviations are specified in the Abbreviations section.

**Table 4:** The roles and mechanisms of lncRNAs in DR.

LncRNAs	Expression Levels in DR	Functions in DR	Target	Reference
CytB	Downregulated in the HG-induced retinal microvessels	Increases ROS production and drives mitochondrial dysfunction	cytochrome B complex III	[108]
MALAT1	Upregulated in the retinas of diabetic patients and mouse models, as well as in retinal endothelial cells under HG	Accelerates retinal vessel impairment and inflammation	p38 mitogen-activated protein kinase (MAPK)	[111]
MEG3	Downregulated in the retinas of DR rat and HG-treated microvascular endothelial cells obtained from retina	Inhibits EndMT	PI3K/Akt/mTOR	[113]
OGRU	Upregulated in the retinas of diabetic patients and mouse models, as well as in HG-treated Müller cells	Accelerates inflammation	miR-221/USP14	[22]
SNHG7	Downregulated in the HG-treated HRMECs cells	Suppresses EndMT and tube formation in HRMECs	miR-34a-5p/XBP1	[105]
VEAL2	Downregulated in the choroid tissue of DR patients	Maintains endothelial permeability	PRKCB2	[109]

## Abbreviations

ANRIL	Antisense non-coding RNA in the INK4 locus
ASMER-1/2	Adipocyte-specific metabolic-related lncRNA 1/2
betaFaar	β cell function and apoptosis regulator
BGLs	Blood glucose levels
BSA	Bovine serum albumin
CaM	Calmodulin
CASC2	Cancer susceptibility candidate 2
CDKN2B-AS1	Cyclin-dependent kinase inhibitor 2B antisense RNA 1
ceRNA	Competitive endogenous RNA
circRNAs	Circular RNAs
DC	Diabetic cataract
DCM	Diabetic cardiomyopathy
DLX6-AS1 (Dlx6-os1 in mice)	distal-less homeobox 6 (DLX6) antisense RNA 1
DN	Diabetic nephropathy
DR	Diabetic retinopathy
ECM	Extracellular matrix
EGR1	Early growth response factor 1
EndMT	Endothelial–mesenchymal transition
ER	Endoplasmic reticulum
EMT	Epithelial–mesenchymal transition
ETC	Electron transport chain
EZH2	Zeste homolog 2
ERAD	ER-associated degradation
FOXA1	Forkhead box A1
GAS5	Growth stabilization specific transcript,
GLIPR2	Glioma pathogenesis related-2
Glis2	ENSMUST00000122896
Gomafu	also referred to as RNCR2/MIAT
HFD	High-fat diet
HG	High glucose
HGMCs	Human glomerular mesangial cells
hnRNPA1	Heterogeneous nuclear ribonucleoprotein A1
IGF2BP2	Insulin like growth factor 2 mRNA binding protein 2
IR	Insulin resistance
KO	Knockout
MALAT1	Metastasis-associated lung adenocarcinoma transcript 1
MDA	Malondialdehyde
MEG3	Maternally expressed gene 3
MGC	Megacluster of nearly 40 microRNAs and their host long non-coding RNA transcript
MME	Membrane metallo-endopeptidase
NEAT1	nuclear paraspeckle assembly transcript 1
NLRP3	NOD-like receptor thermal protein domain associated protein 3
OS	Oxidative stress
PEG	Paternally expressed genes
PRKCB2	Protein kinase C beta 2
PVT1	Plasmacytoma variant translocation 1
ROS	Reactive oxygen species
RTN1	Reticulon-1
SGK1	Serum- and glucocorticoid-regulated kinase 1, Sirt1, Sirtuin1
SHGL	Suppressor of hepatic gluconeogenesis and lipogenesis in mice, B4GALT1-AS1 in human
SNHG14	Small nucleolar host gene 14
SOX6/4	SRY-related high-mobility-group box 6/4
SREBP-2	Sterol regulatory element binding protein 2
STZ	Streptozotocin
TRAF3IP2	Tumor necrosis factor receptor-associated factor 3 interacting protein 2
TUG1	Taurine upregulated gene 1
TUNAR	TCL1 upstream neural differentiation-associated RNA
TXNIP	Thioredoxin-interacting protein
T1DM	Type 1 diabetes mellitus
T2DM	Type 2 diabetes mellitus
UPR	Unfolded protein response
WAT	White adipose tissue
WNT2B	Wingless-type family member 2B
WT1	Wilms tumor protein 1
XIST	X inactive specific transcript
ZEB1	zinc finger E-box binding homeobox 1
ZFAS1	ZNFX1 antisense RNA 1
